# Vision-Based Recognition of Human Motion Intent during Staircase Approaching

**DOI:** 10.3390/s23115355

**Published:** 2023-06-05

**Authors:** Md Rafi Islam, Md Rejwanul Haque, Masudul H. Imtiaz, Xiangrong Shen, Edward Sazonov

**Affiliations:** 1 Department of Electrical and Computer Engineering, The University of Alabama, Tuscaloosa, AL 35487, USA; mislam24@crimson.ua.edu; 2 Department of Mechanical Engineering, The University of Alabama, Tuscaloosa, AL 35487, USA; mhaque2@crimson.ua.edu (M.R.H.); xshen@eng.ua.edu (X.S.); 3 Department of Electrical and Computer Engineering, Clarkson University, Potsdam, NY 13699, USA; mimtiaz@clarkson.edu

**Keywords:** staircase detection, intent recognition, YOLOv5, gradient boost classifier

## Abstract

Walking in real-world environments involves constant decision-making, e.g., when approaching a staircase, an individual decides whether to engage (climbing the stairs) or avoid. For the control of assistive robots (e.g., robotic lower-limb prostheses), recognizing such motion intent is an important but challenging task, primarily due to the lack of available information. This paper presents a novel vision-based method to recognize an individual’s motion intent when approaching a staircase before the potential transition of motion mode (walking to stair climbing) occurs. Leveraging the egocentric images from a head-mounted camera, the authors trained a YOLOv5 object detection model to detect staircases. Subsequently, an AdaBoost and gradient boost (GB) classifier was developed to recognize the individual’s intention of engaging or avoiding the upcoming stairway. This novel method has been demonstrated to provide reliable (97.69%) recognition at least 2 steps before the potential mode transition, which is expected to provide ample time for the controller mode transition in an assistive robot in real-world use.

## 1. Introduction

With the rapid aging of the population, mobility impairment is becoming an increasingly challenging health problem in the United States [1]. People may suffer from impaired ability of ambulation in daily living due to a range of reasons, including limb loss [2], age-related muscle strength decline [3], and neuromuscular pathologies (e.g., stroke) [4]. Motivated by this challenging problem, a variety of wearable robots have been developed to restore lost lower-limb functions (for amputees) (e.g., Ref. [5]) and provide motion assistance to supplement users’ lower-limb joint efforts [6].

As a wearable robot is directly coupled with the user’s limbs and joints, providing coordinated motion or motion assistance based on the user’s motion intent is extremely important. However, recognizing the user’s motion intent in complex real-world environments is difficult. The majority of existing methods rely on mechanical sensor signals (joint angle/velocity, foot pressure, etc.) [7] or muscle activation signals (measured through electromyography) [8] to deduce the intended motion. Such deductive methods tend to suffer from multiple significant issues, such as low accuracy and long delay, as their inputs have been limited to the physical and/or physiological signals extracted from the user himself/herself. Without access to the information on the environment, these intent recognition methods may only react to the user’s actions (which, in turn, are reactions to the upcoming environmental features such as staircases) and are thus unable to predict the user’s intended motion to obtain smooth mode transitions in locomotion.

Motivated by this problem, multiple researchers investigated the use of vision-based environment sensing for wearable robot control. Laschowski et al. developed the ExoNet, an open-source database of high-resolution images of human walking environments [9]. Using such imagery information, environment recognition systems have been developed, which may serve the purpose of wearable robot control (e.g., Ref. [10]). On the other hand, how to use environmental information for motion intent recognition still remains an open question. As a typical example, when an individual approaches a staircase, s/he may still choose to avoid the staircase (i.e., not to go upstairs). Recognizing such motion intent in an accurate and timely manner is critical for the control of wearable robots.

**Figure 1:**
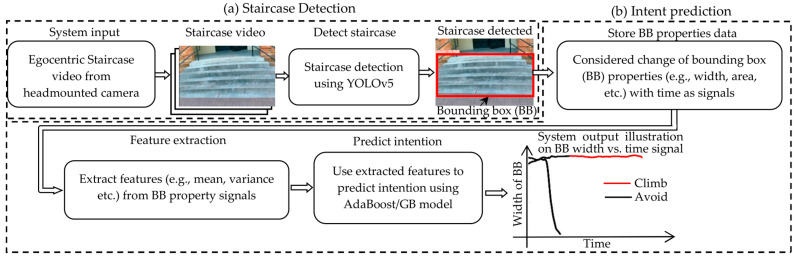
The proposed system comprises two parts. (**a**) Staircase Detection: The system takes egocentric video from a head-mounted camera as input. Then, YOLOv5 model is utilized to detect staircases in the videos and generate and store bounding box (BB) information around them. (**b**) Intent Prediction: The change of BB properties (e.g., width, area, etc.) with time is treated as signals. Then, features (e.g., mean, variance, etc.) are extracted from these signals. Finally, AdaBoost/Gradient Boost (GB) algorithm is employed to predict the intention (climbing or avoiding the staircase) based on the extracted features. An illustration of the system output is provided using the BB width vs. time signal.

In this paper, the authors present a novel vision-based system for human motion intent recognition, using the staircase approach as a typical scenario. This specific use scenario was chosen due to the ubiquitous presence of stairs in real-world environments, as well as the difficulty of mobility-challenged individuals in stair climbing. The assistance provided by wearable robots constitutes a promising solution to overcome such difficulty [11], and the effectiveness of wearable robot assistance can be quantified with instrumented testbeds (e.g., Ref. [12]). The proposed vision-based intent recognition system consists of two primary modules, including a staircase detection with the You Only Look Once (YOLO) v5 model [13] and a bounding-box-based intent classification algorithm constructed with the AdaBoost and gradient boost (GB) methods. Figure 1 illustrates the overview of the proposed method. Egocentric video obtained via the head-mounted camera is first processed with a YOLOv5 model, which detects the object of interest (stairs). To facilitate the training of the YOLOv5 model, we created a dataset of 12,187 staircase images taken from the egocentric view. Beyond the use in this work, the new dataset is expected to complement existing stairs-related image datasets and improve the performance of future vision systems using egocentric cameras. In the detection of stairs, an intent recognizer identifies the user’s motion intent of climbing or avoiding the stairs, utilizing the bounding box properties of the detected staircases. Intent recognizer was developed with the AdaBoost and gradient boost (GB) methods, leveraging their advantages of fast processing speed, low memory use, and high efficiency. The proposed method was evaluated using videos of approaching and climbing/avoiding stairs in real-world ambulation, displaying good performance in identifying the user’s motion intent.

As the primary contribution, the vision-based intent recognizer in this paper, to the best of our knowledge, is the first work that explicitly identifies the user’s intention when interacting with the environment and its key elements (such as a staircase). In existing works (such as [14]), after the vision system detects an important environmental element (stairs as a typical example), the user is expected to engage the element by default (i.e., ascending or descending stairs). In real life, people may still choose to avoid the element (i.e., not to ascend or descend the stairs), and the potential misclassification of the user intent may significantly affect the assistive devices’ control performance and increase the risk of falls. To address this problem, the intent recognizer in this paper explicitly deduces the user’s motion intent when approaching a significant environmental element (such as a stairway) using egocentric images obtained from the vision system. Egocentric images, compared with images from the chest or waist-mounted cameras, better represent the user’s focus of attention and thus serve as a better indicator of his/her motion intent. The proposed intent recognizer extracts bounding box features from the egocentric images and classifies the user intent using an AdaBoost/Gradient Boost classifier. The method is simple to implement and can be easily adapted to other environmental elements (e.g., doors and chairs). Further, as the proposed intent recognizer uses images from the vision system as the sole input, it can work in conjunction with all types of assistive devices (prostheses, exoskeletons, etc.) and motion controllers (impedance control, torque control, etc.). This may become an important building block of wearable robot control systems to improve the robots’ performance and functionality in real-world use.

For the development of this novel method of intent recognition, the research generated a number of technical contributions, including (1) the establishment of the vision-based motion intent recognition framework comprising an environment-sensing module and an intent recognition module; (2) developing the YOLOv5 staircase detection model; (3) identifying the most significant features of the bounding box that can help in detecting not only the intention of staircase engagement/avoidance but also other related intentions/decisions such as how to avoid crossing pedestrians; (4) lastly, a robust and highly accurate simple classification model for detecting the intention to climb or avoid stairs without heavy computational load.

## 2. Staircase Detection

Identifying a staircase to prepare for subsequent climbing motion is one of the fundamental challenges for the control of mobile robots and unmanned ground vehicles [15]. Lower-limb prostheses and assistive devices use different actuator control algorithms in different walking conditions, such as walking, running, stair climbing, and so on, to provide the most convenience to the user [16,17]. So, to choose an appropriate control algorithm, it is crucial to detect the object, such as the staircase, and the user’s intention to climb or avoid that staircase before the activity occurs.

However, predicting the wrong intention can result in an improper control algorithm and wrong actuator control, compromising the user’s safety. So, improving the accuracy of user intention recognition is a very active research field nowadays [18,19,20]. Additionally, combining environmental information with the prosthesis or assistive device user’s walking biomechanics or muscle activation improves intention classification accuracy and allows for more robust control systems [16,21,22]. Thus, when a prosthesis or assistive device user approaches a staircase, for robust decision-making on user intention, the prosthesis or assistive device first has to identify the staircase and then classify the user’s intention of either climbing or continuing usual ground walking.

Many existing approaches extract stair edges from RGB images to detect staircases [23,24,25]. Some use depth images to include range information and make the staircase detector model more robust [15,26]. The problem with line-extraction-based methods lies in the detected straight lines’ reliability and the hypothesis that staircases are always parallel lines. Additionally, in sunlight, most depth cameras, such as Kinect, do not work until the stair is just a few feet apart from the camera [27]. Considering these drawbacks, many researchers have applied deep learning in staircase detection, and these methods have demonstrated better performance [15,28].

However, deep-learning-based methods require a vast amount of data to train the staircase detector model so the model can be generalized properly. Still, to the best of the authors’ knowledge, no annotated dataset contains more than 10,000 RGB images of staircases. Additionally, it is necessary to test the intent recognition model on a dataset that simulates a user approaching a staircase and then climbing or avoiding it. Again, to the best of the authors’ knowledge, a dataset such as that does not exist either. These limitations inspired us to create two datasets. The first consists of 12,187 still staircase images (640 × 480 resolution) annotated in YOLOv5 format and taken from an egocentric perspective. Most of the existing stairs-related images are taken at lower heights (e.g., using chest-mounted cameras); our new dataset complements existing datasets and may improve the performance of vision systems using head-mounted cameras (e.g., cameras embedded in eyeglasses such as Google Glass). The second dataset consists of 375 egocentric videos taken with a head-mounted camera when the user approaches a staircase. This comprehensive staircase-approaching dataset covers a variety of approaching directions (approaching a staircase from the left/right side or in approximately the same direction as the staircase) and motion intents (ascending/descending stairs or avoiding the staircase). From still images of stairs, this dataset of videos exemplifies the visual perception of a human when approaching a staircase and making the related decision (engage/avoid) in real-world ambulation scenarios. Such data may be very useful to computer vision and wearable robot researchers in the development and testing of vision-based intent recognizers for wearable robots. It may be used as a typical example of a human’s visual perception of the environment when interacting with an important environmental element and thus serve as a basis for future expanded datasets that cover a wider variety of real-life scenarios when interacting with other types of important environmental elements (such as doors and chairs).

### 2.1. Training YOLOv5 for Staircase Detection

Considering the intent recognizer’s target application of real-time control of wearable robots, we selected YOLO as the method for detecting stairs, leveraging its very fast speed of processing [29]. Specifically, we used YOLOv5 in the development of the staircase detector to obtain high accuracy of detection, which is highly important for the control of wearable robots and assistive devices [13].

For training the YOLOv5 model, we annotated 12,187 still images of staircases using the ‘imageLabeler’ application in MATLAB and converted the annotations to the YOLOv5 image annotation format.

We augmented the annotated images to increase variability and the number of images. By default, YOLOv5 applies different augmentations on the training images, such as changing hue, saturation, value, image rotation, translation, shear, and mosaic [30]. The rotation and sheer augmentation values were ±15°, and default values were used for the rest of the augmentations [30].

In real-world scenarios, when we approach a staircase with a camera, sometimes the camera goes out of focus and makes the video blur. Additionally, image brightness and contrast differ considerably. Considering these situations, apart from the YOLOv5 augmentations, we applied blur, exposure, and noise augmentations. Out of 12,187 annotated staircase images, we used 80% or 9750 images for training and the rest for validation. After applying the three aforementioned augmentations, the total number of training images became 29,250.

We trained the YOLOv5s, YOLOv5l, and YOLOv5x models with pretrained weights for 500 epochs with a batch size of 8, SGD optimizer, and patience of 20 epochs. The models were pretrained on the MS COCO dataset [31].

### 2.2. Testing YOLOv5 for Staircase Detection

To detect user intention for climbing or avoiding the staircase, we needed videos that show approaching the staircase and climbing or avoiding the staircase. So, based on how a person will approach a staircase, we divided the videos into three main categories:

1. Heading straight to the stair
2. Heading from the left side of the stair
3. Heading from the right side of the stair

**Figure 2:**
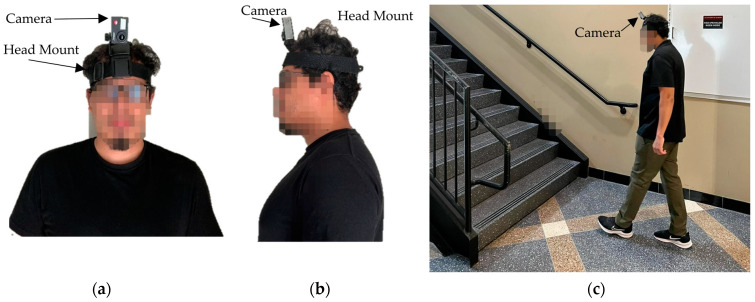
Camera setup for egocentric video capture: (**a**) front view, (**b**) side view, and (**c**) two-point perspective view.

These main categories were divided into five subcategories: one instance was climbing, and the rest were avoiding the staircase. So, there were 15 videos for each staircase. Finally, 375 videos were taken from 25 staircases inside the University of Alabama to create the testing dataset. All the videos were collected at 30 FPS using a head-mounted camera (Campark X25) oriented in portrait mode. Figure 2 illustrates the head-mounted camera setup for capturing egocentric videos.

Then, we tested the previously trained YOLOv5 models on these staircase videos and stored the bounding box (BB) properties of the detected staircase. As YOLOv5x displayed the best detection capability, we used that model’s predicted BB properties for classifying stair climbing or avoiding intention detection.

## 3. Human Intent Recognition

For intention detection, a common practice is to use electromyography (EMG) collected from the residual limb and inertial measurement units (IMUs) [32,33,34,35]. However, these methods are highly user-dependent, and the signals are typically delayed, resulting in delayed prediction [33]. On the other hand, vision-based intention detection methods are mostly user-independent [36]. However, all these methods typically depend on online adaptation and dataset expansion for accurate intention detection [20]. Additionally, they are trained only on a subset of possible sensor data related to all possible prosthesis configurations [20]. Thus, even with large datasets and complex deep learning-based classifiers, it is not guaranteed that the existing models would detect intentions in all possible real-world situations. These shortcomings motivated us to develop a simple linear classifier that does not depend on the user; instead, the classifier uses the properties of the bounding box of the detected object and classifies the user’s intention of either engaging or avoiding it. We hypothesize that when a person approaches an object in the environment (in our case, staircases), the properties, i.e., width, centroid coordinates, and area of the bounding box, increase to some extent. Suppose the person engages that object (climbing the staircase). In that case, the bounding box properties go close to the maximum, and if that person avoids the staircase (continues ground walking), the property of the bounding box goes to a minimum and vanishes at some point. Thus, through extracting features from those bounding box properties in the time domain, we should be able to detect the user’s intention using a linear classifier.

### 3.1. Bounding Box Property Extraction

When the YOLOv5 model detects the staircase in the test videos, it provides four features of the bounding box surrounding the staircase. The four features are the normalized bounding box centroid coordinates along the horizontal and vertical axis and the normalized width and height of the bounding box. Through observing the bounding boxes in the detected videos, we realized the area of the bounding box could be another essential property to detect the user’s intention. So, from the normalized height and width of the bounding box, we also calculated the area of the bounding box.

In some frames of a staircase video, the YOLO model detected more than one bounding box. In those cases, we took the highest height, width, and average centroid value. For the area, we calculated the total area using the following equation:

*A_R_*_1_ = *W_R_*_1_ × *H_R_*_1_ (1)

*Total area* = *A_R_*_1_ + *A_R_*_2_ − *A_I_* (2)

Here, *A*_*R*1_ and *A*_*R*2_ represent the area of the first and second rectangles, *W*_*R*1_ is the width of the rectangles, *H*_*R*1_ is the height of the first rectangle, and *A_I_* is the intercepting/overlapping area between the rectangles.

*W_R_*_1_ = *abs* (*l*1.*x* − *r*1.*x*) (3)

*H*_*R*__1_ = *abs* (*l*1.*y* − *r*1.*y*) (4)

Here, *l*1.*x* is the x-axis coordinate on the left side, and *r*1.*x* is the x-axis coordinate on the right side. *l*1.*y* is the y-axis coordinate on the left side, and *r*1.*y* is the y-axis coordinate on the right side.

Similarly, the second rectangle’s area was calculated. Next, we calculated the intercepting/overlapping area between rectangles using the following equations:

*W_I_* = *min*(*r*1.*x*, *r*2.*x*) − *max*(*l*1.*x*, *l*2.*x*) (5)

*H_I_* = *min*(*r*1.*y*, *r*2.*y*) − *max*(*l*1.*y*, *l*2.*y*) (6)

*A_I_* = *W_I_* × *H_I_* (7)

If *W_I_* or *H_I_* is negative, then the two rectangles do not intersect. In that case, the *A_I_* is 0. With these data, we calculated the total area using (2).

**Figure 3:**
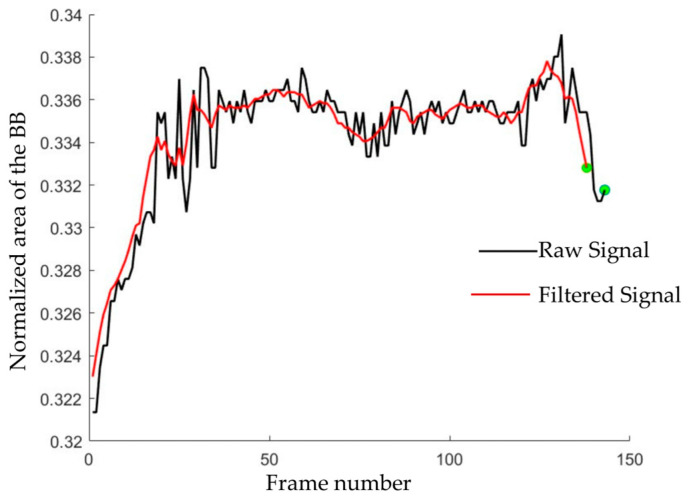
Plot of raw and filtered normalized area of the bounding box vs. frame number. The presence of green dots signifies the conclusion of the signals.

Due to the frame-to-frame fluctuation of the detected bounding box, we observed high-frequency noise in the bounding box property values when plotted against frames. We applied a fifth-order moving average filter to remove the high-frequency noise without compromising the original bounding box properties (Figure 3).

### 3.2. Feature Extraction Bounding Box Properties

We ran a sliding window on the bounding box property plots to get the feature values. In this experiment, we used five different widths of sliding windows and compared them to get the best result. The widths of the windows were 5, 10, 15, 20, and 30 frames. We applied overlapping windows with a stride value of 2 during feature extraction.

**Figure 4:**
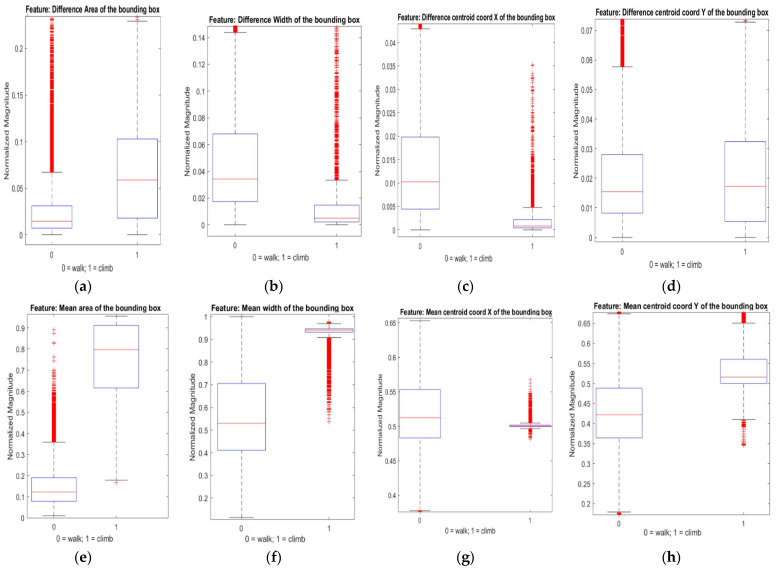
Box plot of features: (**a**) max–min peak difference of the bounding box area; (**b**) max–min peak difference of the bounding box width; (**c**) max–min peak difference of the bounding box X coordinate; (**d**) max–min peak difference of the bounding box Y coordinate; (**e**) mean of the bounding box area; (**f**) mean of the bounding box width; (**g**) mean of the bounding box X coordinate; (**h**) mean of the bounding box Y coordinate.

We extracted simple and less computationally intensive time domain features from the bounding box properties in this paper, i.e., mean and the difference between the maximum and minimum peak of the plots in each sliding window. We extracted the features and plotted box plots to analyze data variability for the two classes. Figure 4 illustrates the box plot of the features for a 15-frame sliding window.

From Figure 4, we observe that the most variability between classes was in the mean area, width, and centroid Y coordinate features. Thus, these features were used to train and test the classifiers.

### 3.3. Intent Recognition Classifiers

Our goal was to prove that computationally inexpensive models can robustly classify user intention from bounding box properties which are user-independent. Additionally, from Figure 4, we observe that the stair climbing and avoidance data have considerable differences that simple classification models can address. So, we trained and compared the results of AdaBoost and gradient-boosted (GB) tree classifiers as they are renowned for their faster training speed, low memory usage, and higher efficiency [37,38].

### 3.4. Dataset for Intent Recognition Classifiers

We annotated the first 35% of each bounding box property data as ‘walk’ and the last 35% of the signals as either ‘walk’ or ‘climb.’ We used the leave-one-out approach for training the classifier. So, all the bounding box property data from one staircase was separated for testing, and the rest of the bounding box property data from the other 24 staircases were used for training the classifiers. After training, the classifiers were tested on the left-out staircase data. This process was repeated 25 times to get results for all 25 test videos.

### 3.5. Training Parameters of the Classifiers

#### 3.5.1. AdaBoost Classifier

The AdaBoost algorithm uses poor learners and adaptively adjusts them through maintaining a collection of weights during the training [37]. We performed a grid search for the optimum parameters of the AdaBoost algorithm. In the grid search, the different numbers of learners were 5, 10, 20, 30, 50, 100, and 200; learning rates were 0.025, 0.05, 0.1, 0.2, and 0.3; and the maximum number of splits were 2, 5, 10, and 20. The optimum hyperparameters for the AdaBoost classifier were as follows: number of learners—50, learning rate—0.05, and maximum number of splits—5.

#### 3.5.2. Gradient Boosting Classifier (GB)

In the GB algorithm, the decision procedure combines the outcome of many weak models to provide a more accurate estimation of the response variable. The principle of this algorithm is to update the new base models in a way that correlates with the negative gradient of the loss function, which represents the whole ensemble [38]. Again, we performed a grid search to find the optimum hyperparameters. The sub-sampling factors tested in the grid search were 0.1, 0.15, 0.5, 0.75, and 1; the different learning rates were 0.025, 0.05, 0.1, 0.2, and 0.3; and the different maximum tree depths were 2, 3, 5, 7, and 10. The best result was found for a sub-sampling factor of 0.15, a learning rate of 0.25, and a maximum tree depth of 2.

### 3.6. Majority Voting for Final Decision

Until now, the proposed approach classified the user intention only based on the features of the bounding box property signal inside the sliding window. Due to noise or other factors such as incorrect object detection, the bounding box features can change rapidly, and as a result, the classifier can repeatedly change its decision. However, to supply a robust control signal to the prosthesis controller, the classifier should not change its decision too often. So, to improve the robustness, we introduced majority voting, i.e., the consecutive classifier decisions voted either climb or walk to decide the final intention. In this study, we experimented with four sets of number-of-classifier predictions to decide the final intention class. The number of votes (predictions) in those four sets were 5, 10, 15, or 30. The class that got the most votes was chosen as the final class. If the classes received an equal number of votes, then the last class that was selected from majority voting was chosen as the final intention.

## 4. Results

First, we compared different YOLOv5 model performances to determine the best model and used the model for further analysis. Next, we determined three performance metrics representing real-world requirements from an intention detector, such as detection time and robustness. Then, we compared the classifier’s performance for different lengths of feature windows. Finally, we compared different voting lengths to get the best possible outcome from the classifiers.

### 4.1. Performance Evaluation of YOLOv5 Models

YOLOv5x is the largest model and had the best performance on the validation set. The next-best was YOLOv5l, and the last was YOLOv5s, the smallest model (Table 1). Thus, we applied YOLOv5x on the test dataset of 375 videos of approaching, climbing, or avoiding a staircase and stored the bounding box properties.

### 4.2. Metrics for Quantifying Intention Classifier Performance

We addressed three aspects of intention detection that are important for the control of an assistive or prosthetic device. First, the intention must be detected some time before the actual intended action occurs so that the operating device has sufficient time to change its mode of operation according to the intended action.

Second, the intention detection must be precise, and misclassification should be as little as possible. Lastly, the classifier should not change its class too often after classifying an intended action. If the classifier changes the detected class multiple times, the prosthesis or assistive device would also switch the mode of operation, and the user would feel discomfort and fall in a worst-case scenario. These three metrics are directly related to user safety, which is one of the prime concerns in a prosthesis design. That is why we addressed misclassification, mean prediction time before stepping on the staircase, and percentage of changes of class while approaching a staircase as performance metrics for our classifier.

**Figure 5:**
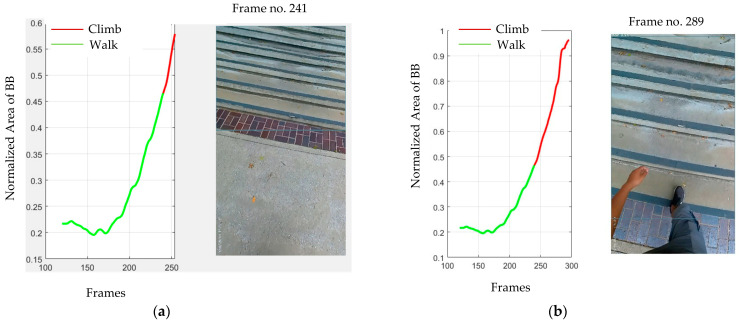
Illustration of calculating intention classification time before stair climb. (**a**) Frame at which intention was detected. (**b**) Frame at which first stepped on the staircase.

To calculate the time between intention classification and action, first, we recorded the time of classification. Second, we recorded the time of the first step on the stairs. We defined the time between these two steps as the prediction time in advance of the action. Figure 5 illustrates those two steps.

Figure 5a illustrates that the intention was detected on frame 241, i.e., 8.03 s (30 FPS), and Figure 5b illustrates the step on the staircase is at the 289th frame, i.e., 9.64 s. So, the intention was classified as about 1.61 s before the intended action occurred.

For the misclassification calculation, if the final prediction from the classifier does not match the actual class, the outcome is classified as misclassification. As we tested using the leave-one-out approach, we added all the misclassifications, divided the sum by the total number of test cases, and obtained the mean misclassification by the classifiers.

Finally, we calculated the percentage of the classifier’s change of decisions after predicting the class for the first time. To calculate it, we added the total number of times the decision was changed and then calculated the percentage to obtain the final percentage of changes of class for climbing or avoiding a staircase.

Table 2 shows a comparison of the classifiers’ performance for different lengths of feature windows. The GB model performs best in all three performance metrics with only 0.45% misclassification. Although AdaBoost has a comparable outcome with GB regarding the misclassification rate and percentage of changes of class in each prediction, GB outperforms AdaBoost in all cases. Next, the feature collected with a 15-frame window performs better with respect to the misclassification rate and the mean percentage of changes of class. The only metric for which the 15-frame signal window lags a bit in performance is the mean prediction time before stepping on the staircase, where features with 30 frames in each window perform better with the GB classifier.

So, after analyzing the results from Table 2, we can say that the GB is the best classifier in intention detection in this study, and the feature window with 15 frames displays the best possible outcome.

**Figure 6:**
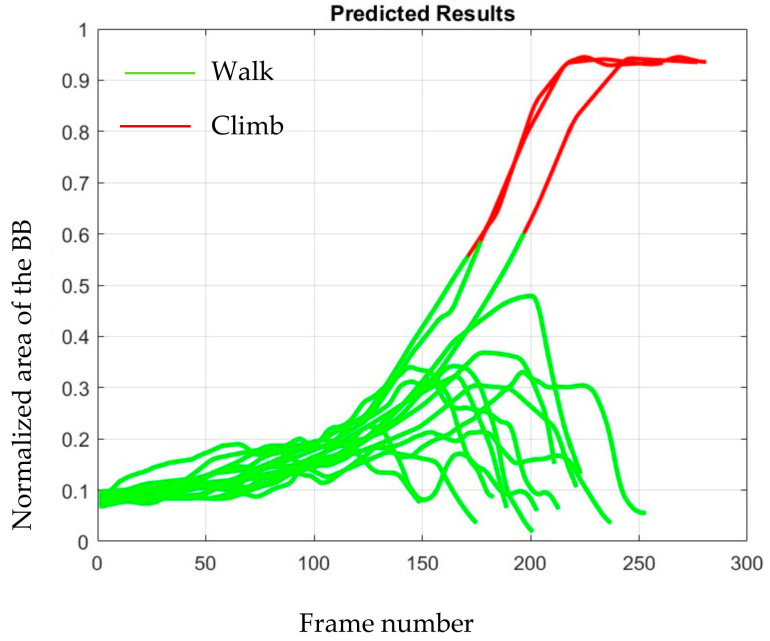
GB classifiers output on a staircase video.

As described above, we applied majority voting to improve the performance even further. Table 3 contains the outcome of majority voting with the different number of votes used for deciding the class. The table shows that although the metrics of mean prediction time before stepping on the staircase and percentage of changes of class are improved with increased votes, the misclassification rate increases slightly. Plotting the GB classifier’s output in one figure on all 25 test staircase videos will obscure it. So, we illustrated the GB classifier’s outcome for one test video in Figure 6, where the feature window had 15 frames and 15 votes were used to decide the class.

## 5. Discussion

Our objective was to develop a robust intent recognition method that can be used to provide reliable detection of the user’s motion intent for the control of wearable robots/assistive devices. Intention recognition from environmental features still requires computationally expensive deep learning models [20,36,39]. We hypothesized that if an object toward which a person is moving can be detected properly with a bounding box, the magnitude of the properties of that bounding box will increase when the person approaches closer to that object and the object would become more prominent to the detector. Then, the features of the bounding box properties can be used to robustly detect the user’s intention.

Vision-based object sensing does not suffer from drawbacks such as low depth sensor range. YOLOv5x is the largest among the YOLOv5 models, contributing to its superior performance on the validation and test datasets (Table 1) [40]. However, the YOLOv5l and YOLOv5s also show promising results, and they can be used to detect staircases on devices with less computational power.

The staircase engagement/avoidance classifier was developed based on the features of bounding box properties. It can be argued that the object detector might be unable to detect the staircase for some frames. Additionally, there can be possible misdetection of objects. Noise such as this can be mitigated through the low-pass filter used before the feature extraction step. Additionally, this method of intention detection does not depend on the user’s gait cycle of other physiological data; instead, the proposed approach uses staircase video collected from a camera. Therefore, we can argue that our approach should be able to detect any user’s intention from any video that shows them approaching a staircase. Thus, this makes the proposed approach user-independent.

The GB model outperforms AdaBoost in all the performance evaluation metrics. The use of loss functions instead of penalizing misclassification and the sensitivity to the outliers of the AdaBoost algorithm might have contributed to the better performance of the GB algorithm. With 15-decision majority voting, the GB model can predict staircase climbing intention about 1.01 s before the actual climbing of the staircase, on average. Its misclassification rate is only 1.37%, which shows that the proposed method not only predicts outcomes about 1 s in advance but also with high accuracy. Additionally, its tendency to change its prediction is only 2.31%, which represents the robustness of the model and proves that this model fulfills the essential requirements in an intention predictor, i.e., it is robust, accurate, and predicts well in advance of the action. In the future, we will research creating an ensemble model to improve the classifier performance even more. If the application of the proposed method requires more accuracy, the user can easily switch to the no-voting option and get accuracy above 99%.

On average, the mean prediction time before stepping on the staircase is higher for 30-frame feature windows. That is because the feature window with 30 frames has more data and more significant features to distinguish between the intention to climb or avoid. Thus, the classifier can distinguish climbing intention early with a 30-frame window, resulting in more time to decide the intention to stair climb before the actual climbing happens.

On the other hand, the mean prediction time before stepping on the staircase decreases with an increased number of votes, as shown in Table 3. This is because an increased number of votes means the algorithm has to wait more time to get a majority vote to decide a class and come to a conclusion. So, more time is lost in getting the decision from the voting and, thus, there is less time before getting the final decision of intention detection. This phenomenon can also explain the decreasing accuracy with increasing voting. In the proposed approach, the accuracy is calculated based on the last frame, whether it is a climb or avoid. Here, misclassified classes play a more significant role when we use 30 votes to decide a class. So, suppose at the end part of a staircase video, the classifier misclassified a class. In that case, there are simply not enough data points left for the classifier to correct the classification, as 30-vote majority voting requires at least 16 votes for deciding a class.

Apart from this, our data suggest that we can make a robust predictor model with simple classifiers and the minimum features of bounding box properties. To the best of our knowledge, this is the first time bounding box properties were used to classify the intention to climb or avoid stairs. In the future, we will research how we can accurately classify intention with even fewer properties of the bounding box while increasing robustness.

As egocentric image-based intent recognition has not been well explored, comparison to existing works is challenging. There are some recent publications on vision systems for human motion/intent recognition. For example, Goncalves et al. developed a deep-learning-based approach for the recognition of human motion (such as walking, stopping, and left/right turn) with an accuracy of 93%, but this method utilizes a walker-mounted RGB-D camera to obtain lower-body motion video, and thus cannot be used for wearable robot control [41]. Darafsh et al. developed a vision system that utilizes videos from stationary-mounted cameras to distinguish people who intend to pass through an automatic door from those who do not [42]. Although this work is also about human intent recognition, its camera placement, data processing method, and desired application all differ significantly from the approach presented in this paper. As such, the work presented in this paper, in the authors’ opinion, represents a major innovation and may significantly improve the performance of wearable robot control systems.

For the future application of the proposed intent recognizer, it can be integrated into a typical hierarchical control system of a wearable robot to identify the user’s motion intent, which will enable the robot’s lower-level controller to calculate the specific control commands for its powered actuators (e.g., desired assistive torque or joint angle trajectory). The intent recognizer does not rely on external sensor signals to function and, thus, can be used in conjunction with all types of wearable robots, such as robotic lower-limb prostheses and assistive ankle/knee orthoses/exoskeletons. The intent recognizer only identifies the user’s motion intent for engaging or avoiding stairs, but the algorithm can be adapted to the recognition of the user’s intent when countering other important environmental elements such as doors and chairs. The limitation is its hardware requirement; as the most popular object detection algorithm, YOLO provides multiple advantages, such as fast processing and high accuracy of recognition. On the other hand, YOLO also requires high-performance hardware, which limits its use in real-time embedded systems. YOLO models have been developed in recent years to enable their implementation in embedded systems (e.g., Fast YOLO [43] and Efficient YOLO [44]) and have been successfully deployed on standalone devices such as the Raspberry Pi 4 [45]; moreover, the integration of a Coral USB accelerator further enhances the detection speed on the Raspberry Pi 4 [46]. In addition, YOLOv5 can also be executed in compact portable devices such as the Jetson Nano, equipped with a high-resolution camera such as the Waveshare IMX477 CSI [47]. Based on such recent trends, we envision that, with fast technological advances in high-performance embedded microprocessors and computer vision, the proposed intent recognizer can be implemented in wearable robot control systems in the near future and significantly improve robots’ performance in navigating real-world environments.

For future work, we plan to incorporate the proposed intent recognizer into complete control systems for wearable robots and characterize its performance in human testing. As most assistive device users are expected to have normal vision (or nearly normal vision after correction), the way they perceive the environment during locomotion should remain largely unchanged, and, thus, we expect the proposed intent recognizer to be applicable to new users without needing much additional training.

## 6. Conclusions

We have created a database of annotated staircase images and trained three YOLOv5 models to detect staircases. We also created a database of 375 videos of 25 staircases, simulating a person walking toward a staircase and avoiding or climbing it. We validated that the properties of the bounding box from a detected staircase can be used to create a simple but robust classifier to classify the intention to climb or avoid stairs. Our data show that the intention detection classifier (GB) is highly accurate (97.69%), and on average, the classifier can detect intention about 1 s before the stair climb. These results suggest that the proposed method can be utilized to send a robust control signal to a prosthesis or assistive device. Our approach also discovered the essential features of bounding box properties for intention detection, leading to future research on intention detection with different types of objects. We also believe our created datasets would help create and test new staircase detection and intention classification models in the future.

## Figures and Tables

**Table 1:** Performance of YOLOv5 models.

Parameters	YOLOv5s	YOLOv5l	YOLOv5x
mAP_0.5 (%)	65.3	78.9	83.4
mAP_0.5:0.95 (%)	28.6	48.2	51.8
Precision	0.71	0.82	0.86
Recall	0.65	0.73	0.74

Here, mAP is mean average precision.

**Table 2:** Performance comparison of the classifier for different feature windows.

Number of Frames in Feature Windows	Percentage of Misclassification		Mean Prediction Time before Stepping on the Staircase (s)		Percentage of Change of Class per Staircase Video	
	AdaBoost	Gradient Boost	AdaBoost	Gradient Boost	AdaBoost	Gradient Boost
5	2.59	1.32	0.68	1.24	23.24	17.38
10	1.86	0.56	0.95	1.51	18.08	11.26
15	1.48	0.45	1.1	1.73	15.29	8.03
20	1.32	0.47	1.33	1.77	14.83	13.04
30	1.45	0.70	1.52	1.94	14.56	13.07

**Table 3:** Results after applying voting on GB Classifier.

Number of Votes for Final Decision	Percentage of Misclassification	Mean Prediction Time before Stepping on the Staircase (s)	Percentage of Change of Class per Staircase Video
No voting	0.45	1.731	8.03
5	0.91	1.383	6.88
10	0.91	1.269	4.82
15	1.37	1.012	2.31
30	1.82	0.662	0.92

## Data Availability

The data presented in this study are available on request from the corresponding author. The data are not publicly available as the article has not been published.
